# Study protocol: a randomised controlled trial testing the effectiveness of ‘Op Volle Kracht’ in Dutch residential care

**DOI:** 10.1186/s12888-015-0498-6

**Published:** 2015-07-21

**Authors:** Martine M. Weeland, Karin S. Nijhof, Ignace Vermaes, Rutger C. M. E. Engels, Jan K. Buitelaar

**Affiliations:** Behavioural Science Institute, Radboud University Nijmegen, P.O. Box 9104, Nijmegen, HE 6500 The Netherlands; Pluryn, P.O. Box 53, Nijmegen, AB 6500 The Netherlands; Karakter Child and Adolescent Psychiatry, P.O. Box 68, Ede, BB 6710 The Netherlands; Trimbos, Utrecht, The Netherlands; Donders Institute for Brain, Cognition and Behavior, Radboud University Medical Centre, Nijmegen, The Netherlands

**Keywords:** Residential treatment, Adolescents, Resilience, Depression, Comorbidity, Mild intellectual disability

## Abstract

**Background:**

Although adolescents are often referred to residential treatment centres because of severe externalizing behaviours, a vast majority demonstrated comorbid symptoms of depression and anxiety. Covert internalizing symptoms in these adolescents might be easily unrecognized and therefore untreated. Adolescents with mild intellectual disability (MID) are overrepresented among youth with both externalizing and internalizing problems. There are yet few treatment programs available for adolescents with both externalizing and internalizing problems.

**Methods/design:**

The CBT-based resiliency program, Op Volle Kracht (OVK), which is based on the US Penn Resiliency Program (PRP), was adapted to suit the needs of adolescents with both externalizing and internalizing problems, either with or without MID, in Dutch residential treatment centres. The effectiveness of this group intervention program of eight sessions will be tested in a randomised controlled trial (RCT) with N = 182 adolescents aged 12–16, allocated to either the target intervention plus treatment as usual (OVK + TAU) or treatment as usual only (TAU). The main outcome variables include depressive symptoms (primary), anxiety, behavioural problems, and group therapeutic climate. Cognitive styles and coping styles will be included as possible mediators. Assessments take place at baseline (T1), one week before the start of the program (T2), immediately after the program (T3), and at three months follow-up (T4).

**Discussion:**

The program assets include its wide implementation possibilities due to low costs, the short duration of the program and the delivery by group care workers, and its suitability for adolescents with MID. Further strengths of the present study design include its robust method (RCT), the ecological validity, and the inclusion of possible mediators of treatment effect. The program emphasizes individual risk factors for depression rather than social and family factors. Implications for practice and future research are discussed.

**Trial registration:**

Dutch Trial Register NTR4836

## Background

In the Netherlands, approximately 25,000 children and adolescents receive residential treatment in the youth welfare system (11,000), youth mental health care (9300) and care for youth with intellectual disabilities each year (5700) [1]. Residential treatment offers 24-h care, including housing, education, and leisure time, in which the daily living environment is used as a therapeutic means. In addition, most institutions offer formal psychological therapy such as cognitive behavioural therapy (CBT) or family interventions, although usually the daily living environment is considered the main therapeutic context [2, 3].

Youth receiving residential treatment are often referred to treatment for severe externalizing behavioural problems (e.g., physical and verbal aggression, disobedience, temper tantrums, hyperactivity, and impulsivity) [4]. Research shows that a large proportion of youth not only have externalizing problems, but also display elevated levels of internalizing problems, such as symptoms of anxiety, depression, and somatization, or have increased risk of developing internalizing disorders such as depressive disorder and anxiety disorder [5, 6]. Moreover, adolescents with both externalizing and internalizing problems more often have MID (e.g. IQ between 50–85) [7, 8]. In these complex cases, more covert, inwardly directed internalizing symptoms may be easily overlooked and given low priority in treatment planning. This might be reflected in treatment outcomes: meta-analyses examining treatment effectiveness show that residential treatment is less effective for internalizing problems than for externalizing problems (effect sizes of .45 and .60, respectively) [9].

Adolescents with comorbid internalizing problems are more severely impaired and have more academic problems, more severe somatic complaints, more problems related to substance use, and poorer overall functioning than adolescents with externalizing problems alone [10–12]. Moreover, youth with a combination of internalizing and externalizing problems have a higher prevalence of suicidal ideation and suicide attempts [13–15]. Longitudinally, comorbid internalizing and externalizing problems are associated with higher rates of adult criminality and higher service utilization rates and costs in adulthood [16]. Thus, underdiagnosis – and therefore undertreatment – of comorbid internalizing problems can lead to further adverse consequences in terms of adolescents’ level of impairment from a long- and short-term perspective.

The high rates of comorbid internalizing and externalizing problem behaviour stress the need for a systematic approach to reduce internalizing problems among youth in residential treatment settings, instead of focusing mainly on externalizing problems. Recent meta-analyses suggest that cognitive-behavioural interventions targeted at emotional resilience for adolescents can significantly reduce internalizing symptoms and decrease the risk for future internalizing symptoms compared to control groups [17–19].

However, most cognitive behavioural interventions are targeted at youth without comorbidities and are insufficiently adapted to youth with complex problems that often are too disorganized in terms of coping with daily life stress, family problems, school problems, etc., to benefit from formal CBT programs. Moreover, to date there are no Dutch CBT programs targeted at youth with MID. This is surprising, given the high rates of youth with MID in residential youth care (>20 %) [1] and the above-average rates of internalizing problems among youth with MID [8]. It is now generally accepted that, due to the social, emotional, and cognitive problems involved, youth with MID require an adapted approach in CBT, including simplified language, smaller steps, and extra emphasis on generalisation to the living environment [20]. Furthermore, most cognitive-behavioural interventions ought to be delivered by specialized health care professionals, such as psychologists and psychiatrists, and therefore do not sort with tendencies in Dutch society toward a more efficient and cost-effective mental health care system with interventions delivered by trained nurses or group workers.

In response to these problems, the existing evidence-based Penn Resiliency Program (PRP) was strongly adapted to (1) suit the needs of the target group, both for adolescents with and without mild intellectual disabilities, and (2) to maximize accessibility by keeping implementation costs low. PRP is a theory-based universal school-based depression prevention program designed for early adolescents (ages 10–14 years) that teaches cognitive-behavioural and coping skills and is one of the most successful programs to increase emotional resilience and to prevent depressive developmental outcomes. Positive results from this program are found in an evaluation of 17 controlled studies evaluating PRP with 2498 participants in total [17]. Recently, PRP has been adapted and translated to fit the Dutch situation, resulting in the creation of the program Op Volle Kracht (OVK, ‘at full strength’). The CBT component of OVK was found to be effective in reducing depressive symptoms in selective samples of girls with elevated levels of depression [21] and adolescents with parents with psychopathology [22]. For the aim of the present study, the OVK program has been adapted to suit the needs of the target group and to suit delivery by nurses and social workers in residential treatment settings.

### Goals and hypotheses

The aim of the present study is threefold: (1) to test the effectiveness of OVK within the usual treatment program (OVK + TAU) in reducing symptoms of depression (primary outcome) compared to treatment as usual alone (TAU), (2) to identify mechanisms that can explain program effects (cognitive bias, coping skills), and (3) to test the effects of OVK on secondary outcomes (anxiety, behavioural problems, and therapeutic climate). It is hypothesized that OVK + TAU will be more effective in reducing symptoms of depression in youth than TAU alone, immediately after the program and at three months follow-up. Regarding the explanatory mechanisms, it is hypothesized that OVK + TAU will decrease negative cognitive biases and increase the adequate coping styles. Both of these constructs are expected to mediate the hypothesized effect of OVK on symptoms of depression. Concerning the secondary outcomes, the OVK program is expected to decrease anxiety, since the program contains cognitive and behavioural strategies that are hypothesized to be effective not only for depression, but also for anxiety (e.g., to recognize and challenge threatening interpretations of neutral events, to employ relaxation strategies [23]). A positive effect on behavioural problems is expected, because behavioural problems are believed to be mediated by deficits in internal impulse regulation and poor self-control [24]. If adolescents learn and adopt cognitive techniques to more adequately regulate their levels of anxiety, they will possess a higher level of self-control to regulate their behaviour, eventually leading to a decrease in problem behaviour. Lastly, the responsiveness of group care workers is hypothesized to increase more in the OVK + TAU group than in the TAU group alone. In the TAU+OVK condition, group care workers will be trained in therapeutic skills necessary to correctly implement the program, including listening skills, conversational skills, and techniques to increase group cohesion. These factors are hypothesized to increase the perceived responsiveness of group care workers.

## Methods

The study design will be reported in accordance with the CONSORT 2010 statement for reporting parallel group randomised trials. The medical ethics committee CMO Region Arnhem-Nijmegen, The Netherlands, has approved the study: NL-49211.091.14.

### Design

**Fig. 1:**
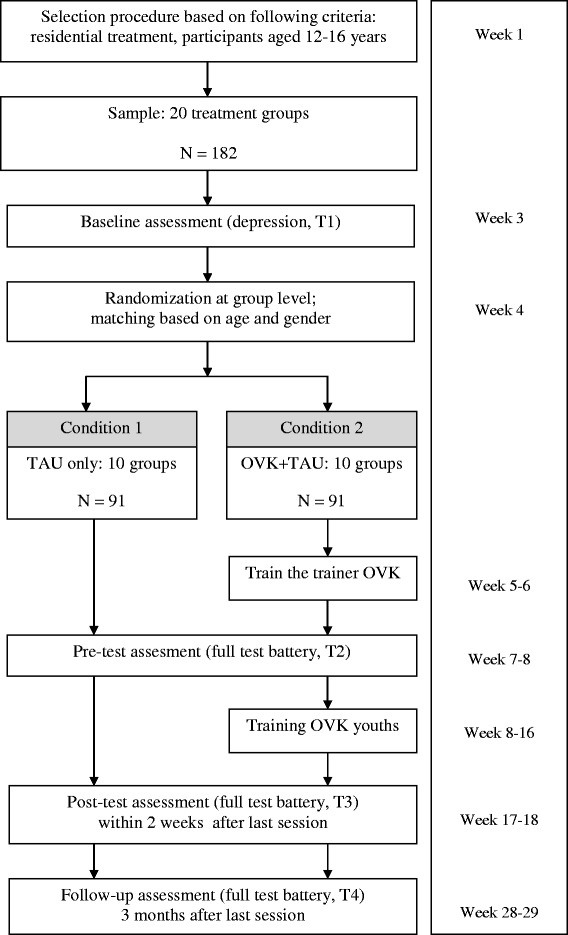
Study design

This study is an open cluster randomised controlled trial with two conditions: the OVK condition incorporated into the usual treatment (OVK+TAU) versus treatment as usual alone (TAU) (see Fig. 1). There is no comparison condition with another program, because (1) there is no Dutch program suitable to increase emotional resilience in a residential group setting, and (2) internationally, there is no evidence-based alternative for the current program. Therefore, the control condition is a correct reflection of ‘treatment as usual’ within residential treatment settings.

Randomization will be conducted by an independent statistician of the Radboud University Nijmegen and will take place on the level of the naturally existing treatment groups rather than on the level of individuals (clustered randomization). In the Netherlands, residential treatment groups usually comprise 8–10 adolescents that follow the same treatment program, live in the same building, and are usually taken care of by a permanent staff of nurses and social workers. The benefits of randomisation of natural groups are as follows: (1) it minimizes contamination among adolescents and staff; (2) it best reflects the natural situation in residential treatment settings, increasing the ecological validity of the study; (3) adolescents who will receive the intervention program know each other already and are used to undertake therapeutic activities together; and (4) staff members who will conduct the program already have a therapeutic relationship with the adolescents. Stratification on the cognitive level (MID versus non-MID) will be needed in order to ensure that the cognitive level is evenly distributed across the control and intervention conditions.

To test the effectiveness of the intervention, all adolescents and their parents will complete self-report questionnaires approximately one month before the start of the program and before adolescents know to which condition they are assigned (Baseline, T1), approximately one week before the start of the program (T2), immediately after the program (T3), and three months after the end of the program (T4). Adolescents in the control condition will complete the questionnaires at exactly the same time points. To make sure that all adolescents (MID and non-MID) understand the questionnaires, adolescents will be assisted in filling out the questionnaires by a trained research assistant that is blind to condition.

The OVK program will be added to the existing treatment program at the participating groups. The 8-week program will be delivered every week by the permanent staff members at the treatment group who has received training specifically for this purpose. By incorporating the program in the standard treatment program at the groups, we maximize the likelihood that all adolescents can attend sessions, and we minimize stigmatization or social exclusion. To minimize attrition rates, adolescents who are dismissed before the end of the program will be given the opportunity to continue the program at the treatment group. The trial is registered at the Dutch Trial Register: NTR4836. The time frame for the data collection will be from October 2014 until March 2015.

### Participants and procedure

An a priori power analysis revealed that a sample of N = 182 would be necessary to obtain a power of .8. Three large institutes for residential treatment, including Pluryn De Hoenderloo Groep (youth care), Pluryn Jan Pieter Heije (care for youth with intellectual disability), and Karakter child and adolescent psychiatry (youth mental health care) will participate in this study. All treatment groups with adolescents between 12 and 16 years old, boys and girls, with a full-scale IQ of 50 or higher will be invited to participate. There are 20 treatment groups with 8–10 adolescents each that meet these criteria, of which are 8 youth care groups (Pluryn De Hoenderloo Groep), 8 groups for youth with MID (Pluryn Jan Pieter Heije), 2 mental health care groups, and 2 mental health care groups for youth with MID (Karakter child and youth psychiatry). Five groups (40–50 adolescents) include only boys, three groups (24–30 adolescents) include only girls, and twelve groups (96–120 adolescents) include both boys and girls. If the participants in the groups agree to participate, they will receive more information about the program and training, and the CDI-2 will be administered as a baseline assessment for depression (T1). Subsequently, groups will be randomly allocated to either one of both conditions. From the treatment groups selected, none of the adolescents will be excluded in order to avoid stigmatization and social exclusion. All parents will receive a letter with information regarding the goals and content of the study and will be asked permission for their child’s participation by passive informed consent. Since not all parents may understand written Dutch, given the expected social and ethnic characteristics of the sample, group care workers will ask the parents if they have understood the letter and if they have any questions. Parents and adolescents are free to withdraw themselves from the study at any time. Next, 2–3 permanent staff members of groups allocated to the intervention condition will receive a three-day training conducted by two experienced and licensed psychologists with broad expertise in cognitive behavioural therapy. Subsequently, T2 will be administered, and the training program will start.

### The intervention

#### Program theory

The program OVK is based on the principles of cognitive behavioural therapy [25]. Cognitive behavioural therapy (CBT) aims to alter maladaptive thoughts, feelings, and behaviour by teaching people skills to recognize their automatic negative thoughts and the consequences on their feelings and behaviour, to challenge the validity of their thoughts, and to change maladaptive thoughts into more helpful, adaptive thoughts. If adolescents successfully adopt these skills, their maladaptive self-schemas will no longer be reinforced, thus creating the opportunity to change negative self-schemas into more adaptive, positive self-schemas. In this way, negative life events will no longer trigger depressive self-schemas, making adolescents more capable and resilient when resolving developmental challenges they encounter in the transition into adulthood. Adolescence provides an ultimate opportunity to preventively alter self-schema’s of depression, since cognitive functions and structures are not yet fully developed and still malleable [26].

#### Program structure

The original OVK program (for a description, see [27, 28]) was reviewed in an expert group of scientists and clinicians in the field of residential youth and mental health care, resulting in the following structural adaptations:

- Treatment duration: Given the relatively short treatment periods in residential care, the program was shortened from 16 to 8 sessions of 45 min, including only the CBT component of the original program.
- Integration in existing treatment plans: The program was incorporated in the existing treatment plans by formulating goals for the training that matched treatment plans. To integrate the taught techniques in the therapeutic living environment, adolescents will make an individual implementation plan together with the group care workers (‘signaleringsplan’).
- Fit with the target group: To make the program suitable for a target group of youth with complex problems, the current program contains less verbal and more practical and visual elements (e.g. pictograms, video clips), the text and layout of treatment materials were simplified, and efforts were made to optimize generalizability to daily life (e.g. use of a personal implementation plan, inclusion of parents and supervisors in home work exercises, and more attention to personal experiences of youth). For youth with MID, a parallel version of the program was created, following the suggestions of the expert group and Didden [20]: (1) the MID version contains less verbal and more visual and practical exercises than the non-MID version, and language is simplified; (2) in the MID version, adolescents receive more support from social workers (e.g. help in homework, help in detecting inaccurate thoughts and challenging their thoughts) than in the non-MID version in order to adapt to their cognitive ability and to optimize generalizability to daily life; (3) the trained skills are taught through smaller sub-steps; and (4) techniques that require higher-order cognitive reasoning (e.g. reasoning about a hypothetical situation; reasoning about other people’s thoughts) are eliminated and replaced by more concrete and behaviour-oriented techniques (e.g. teaching self-instruction and behavioural alternatives in situations adolescents encountered in real life).

In the final program, adolescents are trained in the following cognitive-behavioural skills:

- to recognize and describe feelings and thoughts
- to detect inaccurate thoughts
- to evaluate the accuracy of inaccurate thoughts
- to challenge inaccurate thoughts by considering alternative interpretations
- to use behavioural techniques to regulate emotions (relaxation exercises, distraction of thoughts).

The sessions included group talks, individual and group exercises, and video fragments. After each session, the adolescents are assigned a homework task that takes approximately 15 min. The program will be delivered by permanent staff members at each location that will receive a 3-day training session covering the theoretical principles of CBT, practical training in CBT skills and therapeutic techniques, and an in-depth covering of the intervention protocol. The training will be delivered by experienced and licensed psychologists with broad expertise in CBT. All staff members will receive a detailed manual of the intervention program, and they are offered the opportunity to contact the research team for additional support during program delivery.

### Measures

#### Descriptive and clinical variables

For all participants, demographics (age, birth country of adolescent and that of parents), information on treatment setting (mental health care, youth care, care for youth with MID), IQ-scores, DSM-IV diagnoses, pharmacological information (medication use and dose) and juridical status (whether and when a child protection measure was imposed) will be retrieved from their treatment charts.

#### Primary outcome measures

The primary outcome will be depressive symptoms as measured by the Dutch version of the Children’s Depression Inventory 2 (CDI-2) [29]. The CDI-2 includes both a self- and parent-report questionnaire containing 28 and 17 items respectively. Each item consists of three sentences describing depressive symptoms graded according to severity, from 0 (symptom not present) to 2 (symptom clearly present). A higher total score means more depressive symptoms. This instrument has good reliability and consistency [30], and although no psychometric data are available for youth with MID, the original CDI shows good reliability in samples of adolescents with MID [31, 32].

#### Mediators

Possible mediators include negative cognitive styles and coping styles.

##### *Negative cognitive styles*

will be measured with the Children’s Negative Cognitive Errors Questionnaire – Revised (CNCEQ-R) [33], which is a revised version of the CNCEQ [34]. The CNCEQ-R consists of 16 items that each describe an ambiguous scenario and a possible negative thought. Adolescents rate whether the described thought corresponds to what they would think on a 5-point scale, from 1 (‘not at all like I would think’) to 5 (‘almost exactly like I would think’). Five subcategories of cognitive errors are defined: personalizing, overgeneralizing, threat conclusion, selective abstraction, and underestimation of the ability to cope. The original version of the CNCEQ has a good internal consistency and test-retest reliability among clinical samples [34]. Little is known about the psychometric qualities of de CNCEQ-R in samples of adolescents with MID.

##### *Coping styles*

will be measured with the Children’s Response Style Questionnaire (CRSQ). This 25-item self-report questionnaire categorizes adolescents’ responses to negative emotions in three subscales: ruminative response, distracting response, and problem solving. Each item describes a possible response and is rated on a 4-point scale, ranging from ‘almost never’ to ‘almost always’. The CRSQ demonstrates moderate levels of internal consistency [35].

#### Secondary outcome measures

##### *Anxiety*

according to adolescents will be measured with the Dutch translation of the Spence Children’s Anxiety Scale (SCAS) [36]. The SCAS includes a self- and parent reported questionnaire, each containing 44-items describing anxiety symptoms on a 4-point scale, from 1 (never) to 4 (always). The scale offers excellent internal consistency, concurrent validity, and sufficient test-retest reliability in both child and adolescent samples [37, 38] Spence, 1998; Spence, Barett, & Turner, 2003).

##### *Behavioural problems*

will be assessed using the Strengths and Difficulties Questionnaire (SDQ) [39] for both parents and adolescents. The SDQ contains 25 items divided over 5 scales measuring emotional symptoms, conduct problems, hyperactivity/inattention, peer relationship problems, and prosocial behaviour. The SDQ is widely used and has good psychometric properties in the Dutch population [40].

##### *Responsiveness of group care workers*

will be assessed using the subscale responsiveness of the Dutch translation of the Prison Group Climate Inventory (PGCI). The subscale consists of 12 items rated on 5-point scales, ranging from 1 = ‘I do not agree’ to 5 = ‘I totally agree’. The overall PGCI-scale with 63 items demonstrates good validity and reliability [41].

### Statistical analyses

The expected effect size for this trial was Cohen’s d = .50. Although in a Dutch study on the effectiveness of OVK among youth with subclinical levels of depression (CDI score >15), the effect size of Cohen’s d = .7 was found [21], we chose a more conservative effect size, because slightly lower levels of depression are expected in our target group, and the effect size is known to be smaller if adolescents have lower baseline levels of depression [17]. An a priori power analysis using G*Power 3.1 [42], for a two-sample *t*-test with a one-sided significance level of 0.05 and equal sample sizes, revealed that a sample size of 102 (51 for each group) is required to obtain a power of .8. To accommodate for the clustering effect, we inflated the sample size with a factor of 1.49 following the suggestions of Campbell et al. [43]. Expecting a drop-out percentage of approximately 20 %, based on the number of adolescents that are expected to be dismissed during the program, the total sample size must be 182 (91 for each group).

All data will be analyzed in accordance with the intent-to-treat (ITT) principle but will also be analyzed separately for the completers only. For the ITT analysis, missing values due to loss of respondents or missing item scores will be handled through multiple imputation using the Markov Chain Monte Carlo method in the statistical package Mplus6 [44]. Missing values will be estimated with logistic regression for categorical variables and ordinary regression for continuous variables.

The hypotheses will be tested with mixed effects linear regression analyses in Mplus6. Because adolescents are nested within groups and groups are nested within locations, a three-level analysis is most appropriate to account for the non-independence of the data. However, this technique requires relatively large numbers of clusters (~25) and members in clusters (~25) [45]. With 3 locations, 20 clusters, and 8–10 members in each cluster, a multilevel analysis is not suited. To correct for the problem of the potential non-independence of the data for the clusters, the TYPE = COMPLEX procedure in Mplus will be used. This procedure corrects the standard errors of the parameter estimates for dependency. These standard errors are unbiased estimates and will be greater in comparison to the standard errors derived from individual level analysis. The effect of location will be tested by introducing dummy variables in the regression equation. We will check for possible baseline differences between the two conditions in background variables (e.g., age, sex, and ethnic background, cognitive capacities) and depressive symptoms. These variables will be entered as covariates in the regression equation.

To explore the mediating role of adolescents’ cognitive styles, coping styles, and therapeutic climate, mediation analyses will be performed using Mplus. The significance of indirect paths will be tested via bootstrap sampling. Moderating effects of age, gender, and treatment setting on the relation between depression and anxiety will be explored by entering the moderating variables as predictors for depression/anxiety. The moderation and mediation analyses will be done for the OVK and TAU conditions separately. Reporting of the results of the study will be in accordance with the CONSORT statement [46].

## Discussion

This study protocol presents a randomised controlled trial testing the effectiveness of an adapted version of the newly developed intervention program OVK on depressive symptoms. The program is based on the PRP-program [17] and is specifically adapted to youth with complex problems in a residential setting and with a parallel version for youth with MID. This target group often shows elevated levels of depressive symptoms or is at high risk for the development of depression. The three main goals of the study are to test the effectiveness of OVK plus treatment as usual (OVK+TAU) of depression compared to treatment as usual (TAU) only, to identify mechanisms that can explain program effects and to test the effects of OVK on secondary outcomes. It is expected that OVK + TAU will be more effective in reducing symptoms of depression in youth than TAU only, immediately after the program and at three months follow-up. Concerning the explanatory mechanisms, it is hypothesized that OVK + TAU will decrease negative cognitive biases and increase adequate coping styles. Both of these constructs are expected to mediate the hypothesized effect of OVK on the symptoms of anxiety and depression. Secondary outcomes include anxiety, behavioural problems, and group therapeutic climate.

### Strengths and limitations

An asset of the proposed study is that it is one of the first studies examining a program to increase emotional resilience in a residential setting and is also the only Dutch CBT program specifically adapted to adolescents with MID. There is a strong need for interventions addressing internalizing problems in this complex target group, given that youth in residential settings often display elevated levels of internalizing problems, but problems are often unrecognized and residential treatment is not sufficiently effective in reducing internalizing symptoms. Other than most RCTs, the present study not only examines effectiveness but also takes into account the possible mediators of change. Moreover, the study has a robust design, with a relatively large sample and assessments including multiple informants, and it takes place in a natural setting to optimize ecological validity and generalizability into real-life settings. Another strength is that the program will be group administered, thus enabling all adolescents in treatment groups to participate and minimizing stigmatization. This also optimizes opportunities for adolescents to implement the skills taught in OVK to their daily lives. Finally, the OVK program contains only eight sessions and takes place at treatment groups where adolescents live, therefore a minimal drop-out rate is expected.

A limitation of this study is the open rather than blind design; as participants and other informants included in the assessments (parents and/or caregivers) are aware of the condition they are in, self-reports may be biased. Nevertheless, research assistants administering the questionnaires are blind to condition. Moreover, participants will be told that the program aims to ‘help them to better deal with difficult situations’, without mentioning the specific effects expected on the outcome variables, thereby minimizing expectation effects. Finally, the last planned follow-up assessment is three months. A longer follow-up period would have offered better chances to map longer-term effects of the intervention; however, then a large drop-out rate would be expected, since the follow-up period would extend the relatively short (6–12 months) period during which adolescents are in residential treatment.

With regard to the program content, a limitation is that mainly individual risk factors associated with depression will be addressed (e.g., negative cognitions, coping styles) as opposed to contextual risk factors like family characteristics, friends, and school situations. However, the program contains elements to teach adolescents how to implement the new skills across different contexts. For example, adolescents will make an implementation plan, with actions and helpful thoughts they can use across different situations to influence their mood. Moreover, the group care workers are strongly engaged in the program and are key persons to maintain contact with parents, family, schools, and leaders of leisure activities.

### Implications for practice

If the adapted OVK program is effective among youth within residential care in improving emotional resilience and in reducing (future) depressive symptoms, this may lead to better general outcomes of residential treatment on general functioning. Comorbid internalizing and externalizing problems are longitudinally associated with a higher prevalence of suicidal attempts and suicidal ideation, poorer academic performance, higher service use rates, and higher criminality rates as compared to youth with externalizing problems alone [10–15]. Moreover, effectively reducing internalizing problems in this target group may also lead to a decrease in externalizing problems, as behavioural problems are believed to be mediated by deficits in internal impulse regulation and poor self-control [24]. If adolescents learn and adopt cognitive techniques to more adequately regulate their levels of anxiety, they will possess a higher level of self-control available to regulate their behaviour, eventually leading to a decrease in externalizing problems as well. Thus, the prevention of depression among adolescents with complex problems benefits adolescents themselves, their social contexts (family, friends), and society on a short- and long-term perspective. In addition, the program has the potential to reach as many residential treatment institutes and at-risk youth as possible, without stigmatizing groups, for the following reasons: (1) The program can easily be incorporated into existing residential treatment programs and can be administered by social workers. In this way, extra costs and efforts associated with implementation are kept to a minimum; (2) The program will be tested in all three residential youth care settings in the Netherlands (i.e. general youth care, youth mental health care and care for youth with MID); (3) An adapted version of the program is available for youth with MID; and (4) the program is designed to be accessible to all adolescents in the age of 12–16 years and has no exclusion criteria.

Finally, this study will contribute to our knowledge about depression and anxiety in this complex target group and about factors that mediate intervention effects (cognitive styles, coping styles), which could serve as a starting point for further improvement of residential treatment programs and programs targeted at emotional resilience.

## Conclusion

Internalizing problems are common among youth within residential treatment centres with and without MID, but are often overshadowed by the more visible and disruptive externalizing problems and therefore pose challenges in treatment and diagnostics. As a result, internalizing problems are often undertreated. This protocol describes a study examining the effectiveness of an adapted version of the Dutch CBT-based program OVK on depressive symptoms of adolescents with and without MID in residential treatment settings. This is the first Dutch study evaluating a group-administered CBT-program that is especially adapted to adolescents with MID. Mediators of the effect are taken into account.

## References

[1] Jeugdzorg Nederland. Zorg en voorzieningen voor kinderen en gezinnen van jeugdzorg, jeugd-GGZ en jeugd-LVB. 2012. http://www.vng.nl/files/vng/vng/Documenten/actueel/beleidsvelden/jeugd/2012/20120120_brochure_Zorg_en_voorzieningen_voor_kinderen_en_gezinnen_van_jeugdzorg_jeugd-GGZ_jeugd-LVB.pdf. Accessed 19 November 2014.

[2] Hair HJ (2005). Outcomes for children and adolescents after residential treatment. A review of research from 1993–2003. J Child Fam Stud.

[3] Van der Helm GHP, Klapwijk M, Stams GJJM, Van der Laan PH (2009). ‘What Works’ for juvenile prisoners. The role of group climate in a youth prison. J Child Serv.

[4] Harder AT, Knorth EJ, Zandberg TJ (2006). Residentiële jeugdzorg in beeld. Een overzichtsstudie doelgroep, werkwijzen en uitkomsten [Residential youth care in the picture: A review of literature regarding target group, process and outcome].

[5] Connor DF, Doerfler LA, Toscano PF, Volungis AM, Steingard RJ (2004). Characteristics of children and adolescents admitted to a residential treatment center. J Child Fam Stud..

[6] Gorske TT, Srebalus DJ, Walls RT (2003). Adolescents in residential centers: Characteristics and treatment outcome. Child Youth Serv Rev..

[7] Simonoff E, Pickles A, Wood N, Gringras P, Chadwick P (2007). ADHD symptoms in children with mild intellectual disability. J Am Acad Child Adolesc Psychiatry..

[8] Dekker MC, Koot HM, Van der Ende J, Verhulst FC (2002). Emotional and behavioural problems in children and adolescents with and without intellectual disability. J Child Psychol Psychiatry..

[9] Knorth EJ, Harder AT, Zandberg T, Kendrick AJ (2008). Under one roof: A review and selective meta-analysis on the outcomes of residential child and youth care. Child Youth Serv Rev..

[10] Nottelmann ED, Jensen PS (1995). Comorbidity of disorders in children and adolescents: Developmental perspectives. Adv Clin Child Psychol..

[11] Lewinsohn PM, Rohde P, Seeley JR (1995). Adolescent psychopathology: III. The clinical consequences of comorbidity. J Am Acad Child Adolesc Psychiatry.

[12] Ezpeleta L, Domènech J, Angold A (2006). A comparison of pure and comorbid CD = ODD and depression. J Child Psychol Psychiatry..

[13] Capaldi D (1991). Co-occurrence of conduct problems and depressive symptoms in early adolescent boys: I. Familial factors and general adjustment at Grade 6. Dev Psychopathol.

[14] Capaldi D (1992). Co-occurrence of conduct problems and depressive symptoms in early adolescent boys: II. A 2-year follow-up at Grade 8. Dev Psychopathol.

[15] Fleming JE, Boyle MH, Offord DR (1993). The outcome of adolescent depression in the Ontario Child Health Study follow-up. J Am Acad Child Adolesc Psychiatry..

[16] Knapp M, McCrone P, Fombonne E, Beecham J, Wostear G (2002). The Maudsley long-term follow-up of child and adolescent depression: 3. Impact of comorbid conduct disorder on service use and costs in adulthood. Br J Psychiatry.

[17] Brunwasser SM, Gillham JE, Kim ES (2009). A meta-analytic review of the Penn resiliency Program’s effect on depressive symptoms. J Consult Clin Psych.

[18] Cuijpers P, Van Straten A, Smit F, Mihalopoulos C, Beekman A (2008). Preventing the onset of depressive disorders: a meta-analytic review of psychological interventions. Am J Psychiatry.

[19] Stice E, Shaw H, Bohon C, Marti CN, Rohde P (2007). A meta-analytic review of depression prevention programs for children and adolescents: factors that predict magnitude of intervention effects. J Consult Clin Psychol..

[20] Didden R, Didden R (2006). Gedragsanalyse en cognitieve gedragstherapie bij mensen met een verstandelijke beperking: Een tussenbalans. [Behavioural analysis and cognitive behavioural therapy for people with mild intellectual disability: an overview]. perspectief: Gedragsproblemen, psychiatrische stoornissen en lichte verstandelijke beperking [In perspective: Behavioural problems, psychiatric disorders and mild intellectual disability].

[21] Wijnhoven LAMW, Creemers DHM, Vermulst AA, Scholte RHJ, Engels RCME (2014). Randomised controlled trial testing the effectiveness of a depression prevention program (‘Op Volle Kracht’) among adolescent girls with elevated depressive symptoms. J Abnorm Child Psychol..

[22] Kindt KCM, Kleinjan M, Janssens JMAM, Scholte RHJ (2014). Evaluation of a school-based depression prevention program among adolescents from low-income areas: A randomised controlled effectiveness trial. Int J Environ Res Public Health..

[23] James AC, James G, Cowdrey FA, Soler A, Choke A (2013). Cognitive behavioural therapy for anxiety disorders in children and adolescents (Review). The Chocrane Library..

[24] Granic I (2014). The role of anxiety in the development, maintenance and treatment of childhood aggression. Dev Psychopathol..

[25] Beck AT, Rush AJ, Shaw BF, Emery G (1979). Cognitive Therapy for Depression.

[26] Steinberg L (2005). Cognitive and affective development in adolescence. Trends Cognit Sci.

[27] Tak YR, Van Zundert RMP, Kuijper RCWM, Van Vlokhoven BS, Rensink HFW, Engels RCME (2012). A randomized controlled trial testing the effectiveness of a universal school-based depression prevention program ‘Op Volle Kracht’ in the Netherlands. BMC Public Health..

[28] Kindt KCM, Van Zundert RMP, Engels RCME (2012). Evaluation of a Dutch school-based depression prevention program for youths in highrisk neighborhoods: study protocol of a two-armed randomized controlled trial. BMC Public Health..

[29] Kovacs M (2012). Children’s Depression Inventory 2 (CDI 2).

[30] Bae Y (2012). Test review: children’s depression inventory 2 (CDI 2). J Psychoeduc Assess..

[31] Heiman T (2001). Depressive mood in students with mild intellectual disability: students’ reports and teachers’ evaluations. J Intellect Disabil Res..

[32] Linna SL, Moilanen I, Ebeling H, Piha J, Kumpulainen K, Tamminen T (1999). Psychiatric symptoms in children with intellectual disability. Eur Child Adolesc Psychiatry..

[33] Maric M, Heyne DA, van Widenfelt BM, Westenberg P (2011). Distorted cognitive processing in youth: the structure of negative cognitive errors and their associations with anxiety. Cog Ther Res.

[34] Leitenberg H, Yost LW, Carroll-Wilson M (1986). Negative errors in children: questionnaire development, normative data, and comparisons between children with and without self-reported symptoms of depression, low self-esteem, and evaluation anxiety. J Consult Clin Psychol.

[35] Abela JR, Brozina K, Haigh EP (2002). An examination of the response styles theory of depression in third- and seventh-grade children: a short-term longitudinal study. J Abnorm Child Psychol..

[36] Spence SH (1997). Structure of anxiety symptoms among children: a confirmatory factor analytic study. J Abnorm Psychol..

[37] Spence SH (1998). A measure of anxiety symptoms among children. Behav Res Ther..

[38] Spence SH, Barrett PM, Turner CM (2003). Psychometric properties of the spence children’s anxiety scale with young adolescents. J Anxiety Disord..

[39] Goodman R (1997). The strengths and difficulties questionnaire: a research note. J Child Psychol Psychiatry..

[40] Goedhart A, Treffers F, Widenfelt B (2003). Vragen naar psychische problemen bij kinderen en adolescenten: de Strengths and Difficulties Questionnaire. Maandblad Geestelijke Volksgezondheid [Journal of Public Mental Health]..

[41] Van der Helm GHP, Stams GJJM, Van der Laan PH (2011). Measuring group climate in prison. The Prison Journal..

[42] Faul F, Erdfelder E, Lang AG, Buchner A (2007). G*Power 3: A flexible statistical power analysis program for the social, behavioral, and biomedical sciences. Behav Res Methods..

[43] Campbell M, Thomson S, Ramsay CR, MacLennan GS, Grimshaw JM (2004). Sample size calculator for clustered designs. Comp Biol Med..

[44] Muthén LK, Muthén BO (2010). Mplus User’s Guide.

[45] Wears RL (2002). Advanced statistics: statistical methods for analyzing cluster and cluster-randomised data. Ac Emerg Med..

[46] Schulz KF, Altman DG, Moher D, Grp C (2010). CONSORT 2010 Statement: Updated guidelines for reporting parallel group randomised trials. Trials..

